# Admixture of Liposomal Bupivacaine and Bupivacaine Hydrochloride for Peripheral Nerve Blocks in Adolescents Undergoing Orthopedic Surgery: An Observational Cohort Study

**DOI:** 10.3390/jcm13247586

**Published:** 2024-12-13

**Authors:** Benjamin P. Fiorillo, M. Stephen Melton, Derek Nelsen, Lisa M. Einhorn

**Affiliations:** Department of Anesthesiology, Duke University Medical Center, DUMC 3094, Durham, NC 27710, USA; benjamin.fiorillo@emoryhealthcare.org (B.P.F.);

**Keywords:** local anesthetics, liposomal bupivacaine, regional anesthesia, pain management, pediatrics

## Abstract

**Background/Objectives**: In 2021, the Food and Drug Administration approved liposomal bupivacaine injectable suspension for single-dose infiltration in patients ≥ 6 years of age. Liposomal bupivacaine and bupivacaine hydrochloride admixtures may also be administered off-label for pediatric regional anesthesia including peripheral nerve blocks (PNBs). This single-injection, long-acting technique is not well described in pediatrics but may have benefits over traditional continuous catheter-based systems. The purpose of this investigation was to examine an adolescent cohort who received liposomal bupivacaine/bupivacaine hydrochloride PNBs for orthopedic surgery. **Methods**: Patient, surgical, anesthetic, block characteristics, and post-anesthesia care unit (PACU) outcomes were retrospectively reviewed from February 2020 to June 2024. From February to December 2022, a short follow-up survey was conducted to assess post-discharge patient-reported outcomes. **Results**: There were 524 liposomal bupivacaine/bupivacaine hydrochloride PNBs (106 upper-extremity and 418 lower-extremity) performed in 374 patients with a mean (standard deviation, range) age of 16 (1.2, 11–17) years. Two unilateral PNBs were performed in 150 (40%) patients to ensure an adequate sensory neural blockade. The interscalene (*n* = 81, 15%) and adductor canal (*n* = 140, 27%) blocks were the most common upper- and lower-extremity PNBs, respectively. Intraoperatively, the majority of the cohort (*n* = 258, 69%) underwent monitored anesthesia care (MAC). No patient required conversion from MAC to general anesthesia due to pain. In PACU, 288 (77%) patients reported no pain. Mild and moderate pain were reported by 56 (15%) and 30 (8%) patients, respectively. No patients developed local anesthetic toxicity. The survey results (*n* = 66) indicate that the majority of patients (96%) were satisfied with block analgesia postoperatively. **Conclusion**: Liposomal bupivacaine/bupivacaine hydrochloride PNBs were used successfully in adolescents undergoing a variety of orthopedic surgeries.

## 1. Introduction

Liposomal bupivacaine, or bupivacaine liposome injectable suspension (EXPAREL, Pacira), is bupivacaine, a long-acting amide local anesthetic, formulated in multivesicular liposomes. This preparation increases the local anesthetic duration of action through slow release from the liposome and delays the peak plasma concentration when compared to plain bupivacaine hydrochloride administration [1]. Liposomal bupivacaine is currently approved by the United States Food and Drug Administration (FDA), with limited indications, including interscalene, popliteal sciatic, and adductor canal peripheral nerve blocks (PNBs) in adults and single-dose surgical infiltration of 4 mg/kg for children ≥6 years to <17 years. It is not approved by the FDA for PNBs in children. It is commonly administered as an admixture with bupivacaine hydrocholoride (HCl). Per label instructions, the ratio of bupivacaine HCl to liposomal bupivacaine in milligrams should not exceed 1:2.

Prior to the use of liposomal bupivacaine, postoperative analgesia with a single-injection PNB technique was limited to the local anesthetic duration of action. Traditional, efforts to prolong analgesia relied on adding perineural adjuncts such as clonidine, dexmedetomidine, or dexamethasone, which can increase the duration of analgesia up to 2, 4.5, and 4–8 h, respectively, or using continuous peripheral nerve blocks (CPNBs), involving the placement of perineural catheters and the continuous administration of local anesthetic infusions [2]. CPNBs have limitations for both providers and patients and, importantly, cannot be performed or managed by all providers in all settings. These limitations are particularly relevant for outpatient surgeries within the context of the tremendous growth in the volume and complexity of procedures performed in the ambulatory surgery environment [3]. Thus, the use of a long-acting, single-injection local anesthetic for PNBs that can be administered efficiently and extend the duration of postoperative analgesia is an attractive technique.

For this reason, there have been numerous high-quality randomized controlled trials published on the use of liposomal bupivacaine/bupivacaine HCl PNBs in adults [4,5,6,7,8]. This admixture has been shown to lower “worst pain” scores, decrease opioid use, and increase patient satisfaction in the first week after surgery compared to bupivacaine HCl alone [7,8]. However, this regional anesthesia practice in orthopedic surgery for children remains poorly described, with only three published articles, all involving patients who underwent lower-extremity PNBs. These publications include a single case report (liposomal bupivacaine, *n* = 1), a small case series (liposomal bupivacaine/bupivacaine HCl, *n* = 4), and a non-randomized open-label study comparing liposomal bupivacaine/bupivacaine HCl (*n* = 26) with bupivacaine HCl (*n* = 32) [9,10,11]. Despite the limited data from these investigations, all concluded that the use of liposomal bupivacaine in pediatric PNBs extended pain relief after surgery and resulted in less [11] or no need for [9,10] rescue opioid use postoperatively. Thus, PNBs supplemented with liposomal bupivacaine require further study and may offer a new approach to regional anesthesia for pediatric patients—one that could provide both perioperative and extended analgesia with a single injection.

At Duke University Health System (DUHS), liposomal bupivacaine with bupivacaine HCl has been used off-label for a variety of PNBs for pediatric orthopedic surgery patients since 2020. The purpose of this retrospective study was to identify and characterize a cohort of pediatric patients who received liposomal bupivacaine/bupivacaine HCl PNBs at a single-center, high-volume academic practice. The objectives were to describe the clinical characteristics of patients and their associated PNBs, examine PACU outcomes; and assess patient self-reported post-discharge outcomes.

## 2. Materials and Methods

### 2.1. Ethics Statement

The DUHS Institutional Review Board (IRB) approved this investigation (Pro00110252), which involved conducting a retrospective review of the electronic health records (EHRs) and a prospective research follow-up phone call using an IRB-approved phone script to collect patient-reported outcomes. For those participants who responded to the phone survey, verbal consent for study participation was obtained from parents or legal guardians prior to the commencement of the survey questions.

### 2.2. Study Setting

Pediatric surgical patients (<18 years) are managed in a variety of environments within DUHS, including Duke Children’s Hospital and Health Center (DCH, Durham, NC, USA), a hospital outpatient department (HOPD), free-standing ambulatory surgical centers (ASCs), Duke Regional Hospital, and Duke Raleigh Hospital. DCH is a Level 1 trauma center that provides full-service pediatric tertiary and quaternary medical care and contains over 200 pediatric inpatient beds. Each year, close to twelve thousand pediatric patients undergo surgical procedures within DUHS. At all sites, patients undergo preoperative evaluations and are managed in accordance with the American Society of Anesthesiology Practice Recommendations for Pediatric Anesthesia.

### 2.3. Study Cohort

The population included all children less than 18 years of age who received at least one ultrasound-guided PNB using liposomal bupivacaine at all DUHS surgical locations from 1 February 2020 to 30 June 2024. All PNBs in this cohort were performed by fellowship-trained regional anesthesiologists or pediatric anesthesiologists with additional regional anesthesia training. DUHS EHR data were stored in the queryable database contained within the EPIC-based (Epic Systems; Verona, WI, USA) EHR system. EHR data were abstracted using the self-service reporting tool SlicerDicer to identify pediatric patients who received liposomal bupivacaine as charted in their anesthetic records within the study period. Patients who received liposomal bupivacaine for fascial plane blocks and surgical infiltration were excluded using custom filters within SlicerDicer. Each chart identified using the SlicerDicer tool was manually reviewed to ensure appropriateness for inclusion.

### 2.4. Study Design

The DUHS IRB approved this investigation on 14 February 2022. Following IRB approval, data from patients who underwent surgical procedures from 1 February 2020 to 30 June 2024 were extracted from the EHR, as described above in Section 2.3, and retrospectively reviewed. Patients who underwent surgical procedures between February and December 2022 were called within 1 month of surgery and asked about their experience with their PNB(s).

### 2.5. Study Activities

Following identification of the study cohort, patient characteristics, surgery type, PNB type, liposomal bupivacaine dose (mg), bupivacaine hydrochloride concentration (%), use of general anesthesia (GA) and monitored anesthesia care (MAC), intraoperative and PACU opioid requirements, PACU time (minutes) until first opioid rescue analgesia, and PACU pain scores were retrospectively reviewed. Pain scores were recorded on an 11-point numerical rating scale (NRS) or visual analog scale (VAS). An average PACU pain score was calculated for each patient. Pain was defined as no pain (score 0), mild pain (scores 1–3), moderate pain (score 4–7), and severe pain (8–10) [12]. In the case of patients who received more than one PNB with liposomal bupivacaine for the same procedure, each block was counted separately. For patients who underwent surgery from February 2022 to December 2022, a follow-up survey conducted via a research phone call was used to assess patient-reported outcomes. The survey was administered by members of the research team (DN and LME). Prior to the beginning of the survey, formal verbal consent from parents/legal guardians was obtained, and an IRB-approved phone script was used to conduct the interview. Pediatric respondents answered the questions with their parents/legal guardians present on the phone call. The survey included 5 yes-or-no questions about PNB duration (greater than 24 or 48 h), perceived PNB complications, and overall satisfaction with the pain control and duration of analgesia provided by the PNB. The instrument used for the survey is reported in Table S1.

### 2.6. Data Management

Following IRB approval, all retrospective data extracted from the EHR were reviewed and deidentified. Data were continually extracted every 6 months until 30 June 2024. Those patients who were contacted for survey administration had their information deidentified following completion of the survey to ensure adequate protection of patient privacy. All data were stored in a cloud-based storage service through DUHS (Duke Box, Durham, NC, USA) that complies with security and privacy protections for electronic Protected Health Information (ePHI) mandated for Health Insurance Portability and Accountability Act (HIPAA) compliance. At all times, only members of the research team had access to patient data. There were no missing or incomplete data.

### 2.7. Study Analysis

Descriptive statistics were used to summarize the data. Categorical data are presented as numbers (%), and continuous data are presented as either means (standard deviations, SDs) or medians [interquartile range, IQR] based on normal or non-normal distribution of the data, respectively. All summary statistics were analyzed in R version 4.2.3.

## 3. Results

### 3.1. Cohort Characteristics

A total of 374 patients <18 years of age received at least one ultrasound-guided liposomal bupivacaine/bupivacaine HCl PNB during the study period. The clinical characteristics of the cohort are shown in Table 1. Patient age ranged from 11 to 17 years; the mean (SD) was 16 (1.1). Average patient weight was 76 (23) kgs, with a range of 31–177 kg. All patients underwent orthopedic surgery involving upper-extremity (*n* = 102 patients, 27%) and lower-extremity (*n* = 272 patients, 73%) procedures. The most common upper-extremity surgery was shoulder arthroscopy with capsular and ligament repairs. The most common lower-extremity surgeries were foot/ankle surgeries, including calcaneal osteotomies and fracture fixations, and knee surgeries including anterior cruciate ligament repairs and reconstructions.

Intraoperatively, the majority of the cohort (*n* = 258 patients, 69%) was sedated with propofol with a median [IQR] dose of 75 [55–90] mcg/kg/min under monitored anesthesia care (MAC). The intraoperative opioids used included fentanyl, administered to 37 patients (10%) with a median [IQR] dose of 25 [25–100] mcg, and hydromorphone, administered to 15 patients (4%) with a median [IQR] dose of 0.4 [0.2–0.4]. The majority of patients (*n* = 32, 76%) who received intraoperative supplemental opioids were under GA. Two hundred and twenty-four patients (60%) received a single liposomal bupivacaine/bupivacaine HCl PNB, and one hundred and fifty patients (40%) received two different unilateral liposomal bupivacaine/bupivacaine HCl PNBs for the same procedure to ensure adequate sensory neural blockade. No bilateral blocks were performed. Two patients required conversion to GA from MAC due to upper-airway obstruction.

### 3.2. PNB Characteristics

**Figure 1:**
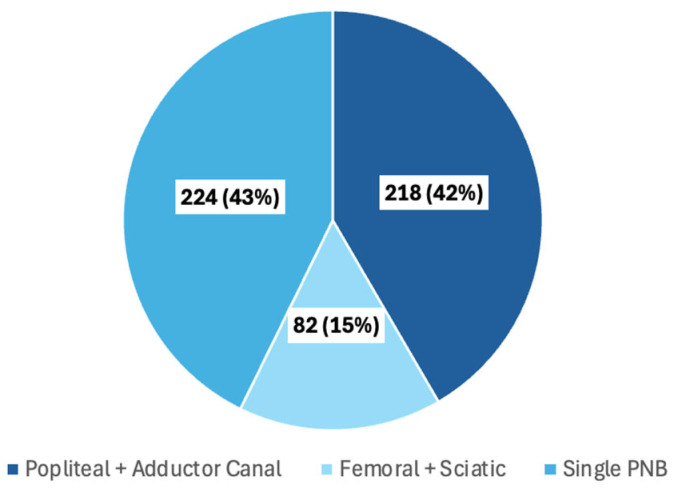
Peripheral nerve block (PNB) combinations, *n* = 524. Data are presented as *n* (%). Figure 1 Legend: The distribution of combination and single PNBs are shown as *n* (%).

A total of 524 PNBs were performed during the study period, 106 in the upper extremities (20%) and 418 in the lower extremities (80%) (Table 2). All PNBs were placed with ultrasound guidance. Of the 524 PNBs, 300 (57%) were performed in combination with a second liposomal bupivacaine/bupivacaine HCl PNB for the same procedure (Figure 1). Popliteal–sciatic and adductor canal PNBs blocks represented the most frequent combination of blocks.

### 3.3. PNB Dosing Regimens

All admixtures consisted of bupivacaine HCl with a milligram dose of bupivacaine HCl to liposomal bupivacaine in a ratio of 1:2 or less and a maximum label-directed liposomal bupivacaine dose of 266 mg. All bupivacaine hydrocholoride concentrations were either 0.25% or 0.5%. Beyond these considerations, there were no formal or standardized dosing regimens. Table 3 shows a clinical dosing guide with various weights and calculated maximum doses and volumes of liposomal bupivacaine and bupivacaine HCl.

**Figure 2:**
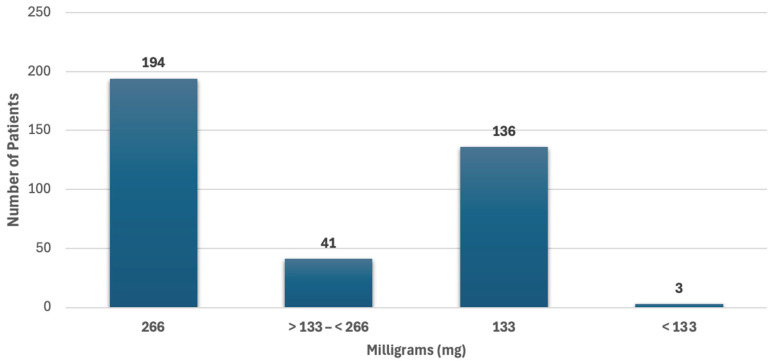
Liposomal bupivacaine doses in milligrams, *n* = 374. Figure 2 Legend. The distribution of liposomal bupivacaine doses is shown.

The liposomal bupivacaine doses administered to our cohort are shown in Figure 2. The majority of patients received doses consistent with the use of one (133 mg) or two vials (266 mg).

Notably, there were 66 (18%) patients (weight range: 37–66 kg) who received a liposomal bupivacaine dose greater than the label-directed weight-based dosing of 4 mg/kg for pediatric patients aged 6 to less than 17 years. The dose range for these patients was 4.1 to 5.6 mg/kg, with a median [IQR] of 4.4 [4.3–4.8] mg/kg. Among those who received a dose > 4 mg/kg, 62 patients (94%) received the maximum 266 mg dose of liposomal bupivacaine. The majority of patients (*n* = 42, 63%) who received a dose > 4 mg/kg had two PNBs for the same procedure, including all 15 patients who received a dose > 5 mg/kg. Based on our experience, Table 4 highlights modifiable factors that may predispose pediatric patients to inadvertently receiving doses exceeding the label-directed dosage.

### 3.4. PACU Outcomes

**Figure 3:**
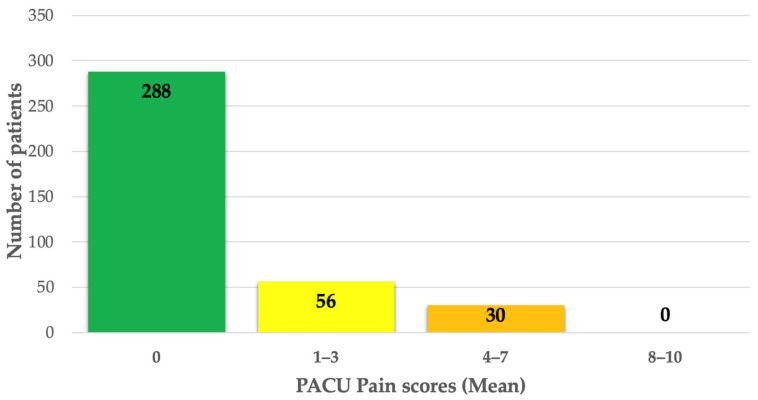
Average patient PACU pain scores, *n* = 374. Figure 3 Legend: The distribution of average PACU pain scores for the cohort is shown. A total of 288 patients (77%) reported no pain in PACU. Mild pain was reported by 56 patients (15%), and moderate pain was reported by 30 patients (8%). No patients reported severe pain.

PACU pain scores and rescue opioid requirements were reviewed. There were 288 (77%) patients who reported no pain in the PACU, 56 (15%) patients who reported mild pain, and 30 (8%) patients who reported moderate pain (Figure 3). No patients reported severe pain in the PACU. All patients who reported moderate pain received opioids in the PACU, including intermittent fentanyl and/or oxycodone. The median [IQR] time until rescue opioid analgesia in the PACU was 26 [16–30] min, with a range of 6–41 min.

### 3.5. Survey Results

The survey was conducted over a period of 10 months from February 2022 to December 2022. There were 66 patients who completed the five-question survey within 1 month of surgery, with a median [IQR] follow-up period of 12 [10–18] days after surgery. The survey respondents underwent 99 liposomal bupivacaine/bupivacaine HCl PNBs and were a representative sample of the total cohort in terms of clinical and PNB characteristics. The majority of survey respondents (*n* = 57, 86%) reported that they experienced analgesia for more than 48 h and were satisfied with the duration of the block (*n* = 60 patients, 91%). Results of the survey are shown in Table 5.

Of the nine patients (four males and five females, with a median weight of 73 kg) who reported PNB duration < 48 h, eight had lower-extremity surgery and one had upper-extremity surgery. The PNBs for these patients were popliteal–sciatic + adductor canal (*n* = 5), popliteal–sciatic (*n* = 1), ankle (*n* = 1), PENG (*n* = 1), and supraclavicular (*n* = 1). There were three patients who reported complications. One patient reported a failed block (a popliteal–sciatic block for calcaneal osteotomy) noted immediately in the PACU, and two patients described post-surgical sensory changes potentially consistent with postoperative neurological symptoms (PONS). The two cases of PONS were reported within 2 weeks of surgery and involved persistent paresthesia along the lateral femoral cutaneous nerve distribution for following a femoral nerve block for anterior cruciate ligament (ACL) repair and persistent numbness at the surgical site for following a PENG block for hip arthroscopy. No falls, hemi-diaphragmatic paresis, persistent laryngeal nerve blocks, prolonged motor blocks, or local anesthetic systemic toxicity (LAST) symptoms were reported. Two patients noted that they perceived that their sensory blocks lasted 5 to 7 days, and two patients reported that their blocks only lasted several hours.

## 4. Discussion

Although there is a growing body of literature on liposomal bupivacaine use in the pediatric population for surgical infiltration and fascial plane blocks [13,14,15,16,17,18,19], there are only a few studies on the use of liposomal bupivacaine/bupivacaine HCl PNBs for children and adolescents undergoing orthopedic surgery [9,10,11]. This observational cohort investigation expands on this sparse literature base by describing and characterizing 574 liposomal bupivacaine/bupivacaine HCl PNBs in the upper and lower extremities of 324 adolescent patients within a single university health system. Our findings over a 4-year period show that this single-injection, long-acting regional anesthetic technique was used successfully for surgical anesthesia and may provide extended post-operative analgesia with high rates of patient satisfaction. The current study expands on the number and variety of liposomal bupivacaine/bupivacaine HCl PNBs in a larger cohort of pediatric patients, including cases involving upper-extremity blocks that have not been previously reported.

We observed a number of important findings that have clinical implications for current practice and also provide a framework for future investigations. In the present study, the population receiving liposomal bupivacaine/bupivacaine HCl PNBs consisted of adolescents with a minimum age of 11 years and a minimum weight of 31 kg. This is significant as most patients were of adult weight and largely received previously established adult dosing regimens. For younger patients (<12 years) with lower weights, there is less information available on safe dosing strategies. This presents an opportunity for future investigations.

Furthermore, it is notable that almost 20% of our cohort received a liposomal bupivacaine dose greater than the label-directed weight-based dosing of 4 mg/kg. LAST is a serious and potentially fatal complication, and there are currently no pharmacokinetic nor safety data on children administered liposomal bupivacaine at peripheral nerve sites. The fact that we did not have an episode of LAST in our cohort does not indicate a presumption of safety. In fact, our findings indicate that it is extremely important for clinicians to be mindful that compared to adults, pediatric patients are more likely to have lower weights and exceed maximum weight-based dosing, especially with a 266 mg dose. In this study, we identified patient-, provider-, and surgery-specific factors and as well as system-level issues that may predispose pediatric patients to inadvertently receiving doses exceeding the label. In our experience, all patients weighed less than 66 kg and were cared for by non-pediatric anesthesiologists in surgical environments that routinely anesthetize both adolescents and young adults, and the majority received two PNBs for the same surgical procedure. From a practice standpoint, liposomal bupivacaine is dispensed in 10 mL (133 mg) and 20 mL (266 mg) vials. Clinicians must calculate the maximum weight-based mg dose allowed for pediatric patients and convert this to the appropriate volume—recognizing that this volume may represent only a partial dose from the vial. Based on our experience at our institution, we recommend the implementation of specific safety practices around ordering liposomal bupivacaine for children; at our center, we now include a “hard-stop” alert in the EHR to ensure that liposomal bupivacaine cannot be ordered from the pharmacy at a dose higher than 4 mg/kg.

In addition, while the decision to induce GA instead of MAC was at the discretion of the attending anesthesiologist, the majority of adolescents were able to tolerate MAC for their surgeries, with minimal to no intraoperative opioid use, and no patient required conversion from MAC to GA due to pain. This indicates a high rate of successful surgical anesthesia with the recommended admixture in this population. Pain in the early postoperative period was well controlled, with no to mild pain for over 90% of the cohort. Finally, there was substantial variability in perceived liposomal bupivacaine/bupivacaine HCl PNB sensory duration, ranging from a few hours to 5–7 days. Further studies examining the duration of action of liposomal bupivacaine/bupivacaine HCl PNBs in pediatric patients may determine if there is more variability in this population.

Perceived complications were noted by three patients in our study. One reported a failed block, one reported persistent paresthesia in the operative extremity, and one reported persistent numbness at the surgical site. A single failed block (1%) is lower than the reported 6–20% PNB failure rate, which is dependent on multiple factors, including provider experience and patient health characteristics [20,21,22]. Perceived complications also occurred in two patients who reported PONS. PONS are multifactorial and as such may be surgical-procedure-, block-, tourniquet-, inflammation-, or position-related or a combination thereof [23,24]. While regional anesthesia has been found to be very safe for children [25], further studies are needed to assess the safety of liposomal bupivacaine/bupivacaine HCl PNBs for this population.

Prior to this investigation, the published literature on the use of liposomal bupivacaine/bupivacaine HCl PNBs in children and adolescents undergoing orthopedic surgery was limited to a case study, a small case series, and a comparative study [9,10,11]. These studies were conducted on pediatric patients undergoing lower-extremity surgery. The case study reported the use of liposomal bupivacaine femoral and sciatic blocks for a 5-year-old who underwent a traumatic amputation of his lower leg [9]. Pain was well controlled for the first 62 h following the blocks. The case series reported successful use of liposomal bupivacaine/bupivacaine HCl adductor canal blocks for three patients undergoing knee surgeries with no post-operative opioid requirements for at least 96 h following the blocks [10]. The comparative study was a retrospective review of prospectively collected data and compared the use of liposomal bupivacaine/bupivacaine HCl adductor canal blocks with 0.25% bupivacaine HCl single-shot adductor canal blocks in 58 patients (liposomal bupivacaine/bupivacaine HCl = 26, bupivacaine HCl alone = 32) undergoing ACL reconstruction [11]. This study reported that a significantly lower percentage of patients in the liposomal bupivacaine/bupivacaine HCl adductor canal block group required postoperative opioids. There was no difference in pain scores over the first 3 postoperative days. Overall, this preliminary study and these case reports support the potential efficacy and tolerability of liposomal bupivacaine use in lower-extremity PNBs for pediatric patients.

Both the prior studies and our 4-year experience highlight that there are numerous potential benefits of a single-injection PNB for children and adolescents. First, block placement takes less time compared to catheter-based PNBs. [26]. Historically, over 90% of pediatric patients had their PNBs placed under GA [25,27]. As single-injection PNBs can be performed faster than catheter-based PNBs, they can often be completed with minimal sedation preoperatively instead of under GA in the operating room (OR), potentially reducing OR time and associated costs. Second, there may be fewer postoperative complications. Single-injection PNBs avoid postoperative catheter-related issues (i.e., leakage, dislodgement, and pump dysfunction), which are the most common complications associated with regional anesthesia in children [25,27]. Third, there are fewer caregiver responsibilities postoperatively. Single-injection PNBs do not require parents or caregivers to be comfortable managing catheter systems at home.

In addition to the benefits associated with single-injection PNBs, there are also considerable advantages of a long-acting PNB, particularly for children and adolescents undergoing orthopedic surgeries. Most notably, there remain ongoing concerns about the well-described phenomenon of “rebound pain”, defined as the unmasking of severe, clinically significant surgical pain following the resolution of the sensory blockade [28,29]. This phenomenon is associated with short- or intermediate-acting single-injection PNBs as the effect of the block dissipates [29,30]. Patient-related factors associated with rebound pain in ambulatory surgery populations include younger age, bone surgery, and female gender [28]. Surgically related factors include complex knee surgeries and shoulder surgery [31]. Rebound pain can negatively impact patient recovery, satisfaction, opioid consumption, and emergency department visits [32,33]. Particularly for adolescents who experience higher incidences of opioid related harms compared to younger children [34], the potential to administer a long-acting (>24 h) analgesic in an ambulatory surgery environment is an important consideration for clinical practice.

Nonetheless, despite its potential benefits for an adolescent population, liposomal bupivacaine is not labeled by the Food and Drug Administration for PNBs in pediatric patients. Due to its patented, branded formulation, randomized controlled trials are challenging to conduct without substantial regulatory and administrative oversight. Efficacy, effectiveness, safety, and cost analysis studies are desperately needed to better inform practice decisions for children undergoing surgical procedures that may benefit from PNBs. Yet, until these additional studies can be conducted, the current investigation provides a retrospective review of a single-institution experience that has pragmatically incorporated liposomal bupivacaine/bupivacaine HCl PNBs into a high-volume academic practice over a 4-year period.

We acknowledge the limitations of this study. This investigation was not designed as a comparative study; thus, we cannot comment on the efficacy of or patient satisfaction with liposomal bupivacaine/bupivacaine HCl PNBs compared to other local anesthetics or catheter systems. In addition, we did not collect information on post-discharge analgesic requirements such as the use of opioids, acetaminophen, or non-steroidal anti-inflammatories. Due to the heterogeneity of the surgical procedures and associated PNBs included, postoperative discharge instructions related to analgesic medications may have varied greatly between patients. Likewise, the follow-up survey was only completed over a 10-month period within a 4-year study, which limits our patient-reported data and may have introduced a sampling and/or reporting bias. Furthermore, we did not conduct a cost-effectiveness analysis to determine if the use of liposomal bupivacaine promotes economic efficiency compared to alternative techniques. Finally, this was an adolescent study, and our experience with this population may not be generalizable to younger children.

## 5. Conclusions

In summary, this retrospective study suggests that liposomal bupivacaine/bupivacaine HCl PNBs may be an alternative technique for providing surgical anesthesia and extended postoperative analgesia to adolescents undergoing orthopedic surgery. Robust comparative investigations are needed to assess the analgesic efficacy of liposomal bupivacaine/bupivacaine HCl PNBs versus bupivacaine HCl single-shot PNBs and catheter based CPNBs. Additionally, further studies focused specifically on safety in pediatrics are necessary to determine the pharmacokinetic properties of liposomal bupivacaine when used at peripheral nerve sites and define the dosing relationship with bupivacaine HCl. Finally, future studies may consider the evaluation of other meaningful clinical outcomes, like postoperative opioid consumption, physical function, return to normal activities, sleep, and cost in age or surgery specific populations.

## Figures and Tables

**Table 1:** Patient, surgery, and anesthetic characteristics.

Cohort Characteristics	Patients (*n* = 374)
Sex	
Male	219 (59%)
Female	155 (41%)
Age (years)	16 (1.2), 11–17
Weight (kg)	76 (23), 31–177
Race and Ethnicity	
White	233 (62%)
Black	105 (28%)
Other	36 (10%)
Hispanic	52 (14%)
Surgery Location	
Main Hospital	102 (27%)
Outpatient Department	187 (50%)
Surgery Center	85 (23%)
Anesthesia Type	
Sedation	258 (69%)
GA	116 (31%)
Sedation to GA	2 (1%)
Surgery Type	
Upper Extremity	
Shoulder	77 (21%)
Arm/Elbow/Hand	22 (6%)
Clavicle	3 (1%)
Lower Extremity	
Knee	121 (32%)
Foot/Ankle	125 (33%)
Hip	23 (6%)
Other	3 (1%)
Number of PNBs	
One	224 (60%)
Two	150 (40%)

Categorial data are presented as *n* (%). Continuous data are presented as means (standard deviation), with range. Kg = kilogram, GA = general anesthesia, and PNB = peripheral nerve block.

**Table 2:** Peripheral nerve block (PNB) characteristics.

PNB Characteristics	PNB (*n* = 524)
Lower Extremity	
Popliteal	122 (23%)
Adductor Canal	140 (27%)
Femoral	94 (18%)
Sciatic	43 (8%)
Ankle	1 (0%)
PENG	18 (3%)
Upper Extremity	
Interscalene	81 (16%)
Supraclavicular	11 (2%)
Infraclavicular	14 (3%)

**Table 3:** Clinical dosing guide for weight-specific liposomal bupivacaine/bupivacaine HCl administration.

Weight (kg)	Max Liposomal Bupivacaine Dose (mg)	Max Liposomal Bupivacaine Volume (mL) *	Max Bupivacaine HCl Dose (mg)	Max Volume of Bupivacaine HCl 0.5% (mL)	Max Volume of Bupivacaine HCl 0.25% (mL)
20	80	6.0	40	8	16
25	100	7.5	50	10	20
33.25	133	10	66.5	13.3	26.6
37.5	150	11.3	75	15	30
50	200	15.0	100	20	40
60	240	18.0	120	24	48
≥66.5	266	20	133	26.6	53.2

* Liposomal bupivacaine = 13.3 mg/1 mL.

**Table 4:** Factors associated with liposomal bupivacaine dosages exceeding 4 mg/kg for pediatric patients.

Factor Type	Modifiable Risk Factors
Patient-specific	1. Weight < 66 kg 2. Two peripheral nerve blocks for same procedure
Surgery-specific	1. Surgery commonly performed on both adolescents and young adults (i.e., including, but not limited to, knee ligament reconstruction; periarticular osteotomy; shoulder arthroscopy)
Provider-specific	1. Non-pediatric anesthesiologist
System-level	1. Mixed practice surgical environments in which adults and adolescents are cared for at the same location 2. Use of surgery-specific regional block protocols, resulting in less individualization of care 3. Lack of pharmacy order sets with dosing safety checks

**Table 5:** Postoperative survey results; *n* = 66.

Response	PNB Duration > 24 h	PNB Duration > 48 h	Satisfaction with PNB Duration	Complications	Satisfaction with PNB Analgesia
Yes	63	57	60	3	63
No	3	9	6	63	3
% Positive	96%	86%	91%	5%	96%

PNB = peripheral nerve block.

## Data Availability

Raw data can be obtained by contacting the corresponding author.

## Supplementary Materials

The following supporting information can be downloaded at https://www.mdpi.com/article/10.3390/jcm13247586/s1. Table S1: Postoperative survey instrument.
