# Supplementary material for: Admixture of Liposomal Bupivacaine and Bupivacaine Hydrochloride for Peripheral Nerve Blocks in Adolescents Undergoing Orthopedic Surgery: An Observational Cohort Study
Source: J Clin Med. 2024 Dec 13;13(24):7586. doi: 10.3390/jcm13247586 (PMC11678512; doi:10.3390/jcm13247586)
Supplement: Supplementary file 1 [file jcm-13-07586-s001.zip › jcm-3327546-supplementary.pdf]

Supplementary Table 1. Postoperative survey instrument.

**Question**	**Response**
1. Do you feel the nerve block lasted longer than 24 hours?	Yes/No
2. Do you feel the nerve block lasted longer than 48 hours?	Yes/No
3. Were you satisfied with the nerve block’s duration?	Yes/No
4. Did you experience any side effects or complications? (persistent numbness, failed block, etc)	Yes/No
5. Were you satisfied with the pain control provided by the nerve block?	Yes/No
